# Intracellular autofluorescence enables the isolation of viable, functional human muscle reserve cells with distinct Pax7 levels and stem cell states

**DOI:** 10.1186/s13287-025-04811-7

**Published:** 2025-12-01

**Authors:** Axelle Bouche, Diego Michel, Perrine Castets, Didier Hannouche, Thomas Laumonier

**Affiliations:** 1 Cell Therapy and Musculoskeletal Disorders laboratory, Department of Surgery, Geneva University Hospitals & Faculty of Medicine, 1 Rue Michel Servet, 1211 Geneva, Switzerland; 2 Department of Cell Physiology and Metabolism, Faculty of Medicine, University of Geneva, Geneva, Switzerland

**Keywords:** Autofluorescence, Human muscle reserve cell, Pax7, Quiescence, Reactivation, Transplantation

## Abstract

**Background:**

Human muscle reserve cells (MuRC) represent a quiescent MuSC population generated in vitro that exhibit heterogeneous Pax7 expression, with a Pax7^High^ subset in a deeper quiescent state. However, the conventional method of identifying Pax7^High^ cells involves intracellular staining, which limits their viability for functional studies. This work investigates whether autofluorescence (AF) could be used as a potential biomarker to identify functionally distinct human MuRC subpopulations.

**Methods:**

Human myoblasts (MB) and MuRC were analysed for AF by fluorescence microscopy and flow cytometry. Cellular metabolic composition was assessed by NADH/NADPH quantification and lipid staining. Human MuRC subpopulations were sorted by AF intensity and analysed for Pax7 expression, cell cycle re-entry, proliferation, clonal expansion, and myogenic differentiation. In vivo transplantation of MuRC-AF^High^ and MuRC-AF^Low^ populations into immunodeficient mice assessed survival and regenerative potential using bioluminescence imaging and immunohistochemistry.

**Results:**

Human MuRC exhibited a threefold increase in autofluorescence intensity compared to MB, with a peak at 405 nm excitation, likely linked to a 1.6-fold increase in lipid content, while NADH/NADPH levels remained comparable. Flow cytometry identified MuRC-AF^High^ as a Pax7^High^-enriched subpopulation, indicative of a deeper quiescent state. Functionally, MuRC-AF^High^ cells showed delayed cell cycle re-entry and slower proliferation yet maintained full differentiation capacity. In vivo, both MuRC-AF^High^ and MuRC-AF^Low^ survived transplantation, contributed to Pax7 + MuSC formation, and retained regenerative potential upon re-injury.

**Conclusion:**

Autofluorescence enables the isolation of distinct human MuRC subpopulations. The AF^High^ subset contains a high proportion of Pax7^High^ cells and shows delayed activation yet retains engraftment efficiency that is comparable to that of the AF^Low^ subpopulation. These findings suggests that AF could be used as a biomarker to identify functionally distinct human muscle progenitor subsets while preserving their regenerative potential for future use.

## Background

Muscle stem cells (MuSC) are essential for skeletal muscle regeneration [1–4]. Following injury, MuSC become activated, proliferate, and differentiate to repair damaged myofibers. In parallel, a subset of proliferating MuSCs undergoes self-renewal to restore the quiescent MuSC pool [5, 6], ensuring long-term regenerative capacity. Intramuscular transplantation of MuSCs has long been explored as a potential strategy to regenerate skeletal muscle after severe trauma or to treat muscular dystrophies. However, clinical trials using myoblasts (MB) or mesoangioblasts have had limited success [8]. There is emerging evidence that MuSCs lose their regenerative potential rapidly when cultured under standard ex vivo conditions, primarily due to premature activation from quiescence [9, 10]. Therefore, preserving MuSC quiescence is essential to maintaining their regenerative capacity [11–14]. We have previously demonstrated that human muscle reserve cells (MuRC), which are generated in vitro*,* constitute a population of quiescent Pax7 + MuSC. These cells have an enhanced ability to regenerate and contribute to the Pax7 + MuSC pool following intramuscular transplantation [15]. More recently, we identified heterogeneity within the MuRC population based on Pax7 expression. This revealed a Pax7^High^ subpopulation in a deeper quiescence state characterized by lower metabolic activity and reduced commitment to myogenic differentiation [16]. Similarly, studies using transgenic Pax7-nGFP mice have shown that the Pax7^High^ MuSC subpopulation possesses superior regenerative capacity [17]. Single-cell transcriptomic analyses of human skeletal muscle further support the presence of a Pax7^High^ MuSC subpopulation, primarily composed of quiescent myogenic cells [18, 19]. These findings highlight the potential of human Pax7^High^ MuRC as a promising stem cell source for therapeutic applications in muscle disorders. However, current methods for isolating Pax7^High^ human MuRC rely on fixation and permeabilization, rendering these cells non-viable and unsuitable for functional studies.

Mammalian cells exhibit intrinsic autofluorescence (AF) due to the presence of various endogenous molecules, such as nicotinamide adenine dinucleotide (NAD), flavins, fatty acids and collagens [20]. The AF spectrum is shaped by specific intracellular compounds and has been used to distinguish subpopulations of cancer stem cell [21–25]. Recent studies also suggest that AF can serve as a label-free marker with which to assess the activation state of neural stem cells [26]. Building on these insights, we investigated whether intracellular AF could be used to non-invasively isolate viable functionally distinct subpopulations of human MuRCs. Our results demonstrate that human MuRC-AF^High^ cells constitute a distinct subpopulation enriched in Pax7^High^ cells, characterized by prolonged quiescence and delayed activation whilst retaining regenerative potential. These findings position AF as a promising biomarker for identifying functionally distinct muscle progenitor subsets and highlight its importance in muscle regeneration research.

## Methods

The work has been reported in line with the ARRIVE guidelines 2.0.

### Human myoblasts isolation and culture

Human muscle biopsies were obtained during orthopaedic surgery from healthy patients with no known muscle disease. Muscle tissue was enzymatically dissociated using 0.05% trypsin–EDTA (Thermo Fisher, St. Louis, MO, USA) and freshly isolated cells were cultured for 5 to 7 days in growth medium (GM). The GM consisted of Ham's F10 (Thermo Fisher, St. Louis, MO, USA) supplemented with 15% fetal calf serum (FCS; Thermo Fisher), bovine serum albumin (0.5 mg/ml; Sigma-Aldrich, St. Louis, MO, USA), fetuin (0.25 mg/ml; Sigma-Aldrich), epidermal growth factor (10 ng/ml; Life Technologies), dexamethasone (0.39 μg/ml; Sigma-Aldrich), insulin (0. 04 mg/ml; Sigma-Aldrich), creatine (1 mM; Sigma-Aldrich), pyruvate (100 μg/ml; Sigma-Aldrich), uridine (50 μg/ml; Sigma-Aldrich) and gentamycin (5 μg/ml; Thermo Fisher). Pure populations of human myoblasts (MBs; CD56 + /CD146 + /CD82 +) were then isolated by flow cytometry using a BD FACS Aria Fusion (BD Biosciences, New Jersey, USA). MBs were expanded in GM for up to seven passages, corresponding to less than 30 cell divisions.

### In vitro generation of human muscle reserve cells (MuRCs)

Human MBs were cultured in GM to 80% confluence and then transferred to differentiation medium (DM) for 60 h. DM is a DMEM-based medium (Life Technologies) supplemented with bovine serum albumin (Sigma-Aldrich; 0.5 mg/ml), epidermal growth factor (Life Technologies; 10 ng/ml), insulin (Sigma-Aldrich; 0.01 mg/ml), creatine (Sigma-Aldrich; 1 mM), pyruvate (Sigma-Aldrich; 100 μg/ml), uridine (Sigma-Aldrich; 50 μg/ml) and gentamycin (Life Technologies; 10 μg/ml). After brief trypsinisation, the myotubes were removed and the remaining cells were passed through a 20 µm pre-separation filter (Miltenyi Biotec, Bergisch Gladbach, Germany) to isolate mononucleated human MuRCs.

### Live imaging microscopy

Freshly isolated human MBs or human MuRCs were plated in GM for 3 h and subsequently imaged using a Zeiss Axio Observer A1 microscope equipped with a Lambda XL illumination system (Sutter Instrument, Novato, CA, USA).

### Flow cytometry analysis and sorting

#### Autofluorescence

Freshly isolated human MBs and MuRCs were resuspended in FACS buffer (PBS—2% BSA—0,02% sodium azide) and incubated on ice for 30 min with a mouse anti-human CD56-PE antibody (BD Pharmigen, cat# 555,516) to exclude non-myogenic cells. After two washes in FACS buffer supplemented with DAPI (1 μg/ml) to exclude dead and apoptotic cells, cells were analyzed and/or sorted using a BD FACS Aria Fusion (BD Biosciences). To optimize AF detection, all available excitation/emission spectra were tested. The FL1 channel (excitation at 405 nm, emission at 450/40 nm) and FL2 channel (excitation at 640 nm, emission at 670/14 nm) were selected for AF analysis and cell sorting based on their ability to maximize fluorescence signal intensity.

#### Lipid droplet staining

Freshly isolated human MBs and MuRCs were incubated with 2 µg/ml BODIPY 493/503 (Thermo Fischer, cat# D3922) for 15 min at 37 °C. Following incubation, cells were washed three times with FACS buffer and stained with a mouse anti-human CD56-PE antibody (BD Pharmigen, cat# 555,516) for 30 min on ice. After three additional washes, cells were kept on ice and analysed using a BD LSR Fortessa (BD Biosciences).

#### Intracellular Pax7 staining

Freshly isolated human MBs, MuRCs, MuRC-AF^High^, and MuRC-AF^Low^ cells were washed twice with PBS and stained for 10 min at room temperature (RT) with Fixable Viability Stain (FVS) 780 (1:1000, BD Biosciences, cat#565,388) to exclude non-viable cells. After two PBS washes, cells were fixed and permeabilized using the Transcription Factor Buffer Set (BD Biosciences, cat#562,574) followed by a 5 min incubation at RT with human TruStain FcX^™^ (BioLegend, San Diego, CA, USA, cat#422,302). Cells were then incubated for 40 min at 4 °C with a mouse anti-human Pax3/7 Alexa Fluor^®^ 647 antibody (Santa Cruz Biotechnology, Dallas, TX, USA, cat#sc-365843), or with an Alexa Fluor^®^ 647-labeled isotype-matched control antibody for negative control samples. After three times with Perm/Wash buffer, cells were resuspended in 300 μl PBS and analyzed using a BD LSR Fortessa (BD Biosciences).

### Quantification of NADH and NADPH levels

NADH and NADPH levels were quantified in human MuRCs and MBs using the NAD/NADH Quantification Kit (MAK037, Sigma Aldrich) and the NADPH Assay Kit (ab186031, Abcam). For NADPH analysis, cells were lysed in the provided buffer (1 × 10^6^ cells/100 µl) prio to the enzymatic reaction. For NAD/NADH quantification, 4 × 10^5^ MBs or MuRCs were pelleted, and NADH/NAD were extracted and deproteinized using 10 kDa cut-off centrifugal filters (UFC501024, Millipore). A volume of 25 µl from each sample was transferred to a 96-well plate and incubated for 1 h at RT with the respective enzymatic reagents. Absorbance was measured at 460 nm (NADPH) or 450 nm (NAD/NADH) using a Sense plate reader (Hidex, Turku, Finland).

### Cell proliferation assay

Freshly isolated human MBs, MuRC-AF^High^, and MuRC-AF^Low^ cells were seeded at a density of 3,000 cells/cm^2^ and cultured in GM for up to 7 days. with medium refreshed every 2 days. On days 4 and 7, cells were trypsinised, washed with PBS, and counted using the CellDrop BF automated cell counter (DeNovix, Wilmington, USA). Population doubling level (PDL) was calculated using the formula: PDL = 3.32 (log(total viable cells at harvest/total viable cells at seeding)).

### Clonogenic assay

Individual human MuRC-AF^High^, or MuRC-AF^Low^ cells were sorted using a BD FACS Aria Fusion cell sorter (BD Biosciences) and seeded into 96-well plates. Cells were cultured in GM for 8 days, with half of the medium replaced every 2 days. Following incubation, cells were stained with Hoechst 33,342 at 20 µg/ml (Thermo Fischer, cat# C10640G) for nuclear visualization, washed with PBS, fixed with PBS, 4% PFA for 10 min at RT. After three additional PBS washes, images of each well were acquired using a Cytation5 imaging reader (Agilent Technologies) and clone size analysis was performed using Gen5 software (Agilent Technologies).

### EdU staining

Human MBs, MuRC-AF^High^, and MuRC-AF^Low^ were isolated by flow cytometry, seeded onto glass coverslips at a density of 3,000 cells/cm^2^ and cultured for 24 h in GM supplemented with 10 µM EdU (Click-iT^™^ Plus EdU Cell Proliferation Kit for Imaging, Invitrogen, cat#C10640). EdU incorporation was assessed at three time points: immediately after sorting (D0; 25′000 cells/cm^2^), one day post-sorting (D1; 25′000 cells/cm^2^) and four days post-sorting (D4; 7′500 cells/cm^2^). Following incuabtion, cells were rinsed three times with PBS, fixed with 4% PFA in PBS for 15 min at RT and washed again three times with PBS. Cells were then permeabilized and blocked in PBS containing 5% goat serum (GS), 0.3% Triton X-100 for 30 min, followed by a PBS wash. EdU detection was performed using the Click-iT^®^ Plus reaction cocktail for 30 min. After final washes, coverslips were mounted onto glass slides using ProLong^™^ Glass Antifade Mountant with NucBlue^™^ (Invitrogen, cat#P36981) and images were acquired using a Zeiss Axio Imager Z1 microscope.

### Myogenic differentiation assay

Freshly isolated MBs, MuRC-AF^High^, and MuRC-AF^Low^ cells were seeded at high density (30,000 cells/cm^2^), cultured in GM for 24 h, and then switched to DM for 48 h. Adherent cells were washed with PBS, fixed in 4% PFA in PBS, for 15 min at RT and rinsed three times with PBS. Cells were blocked for 60 min at RT in PBS containing 5% GS, 0.3% Triton X-100, then incubated overnight at 4 °C with a mouse anti-⍺-actinin (Sigma, cat#A7811, 1:500) and a rabbit anti-MEF2C (Cell Signaling, cat#5030, 1:300), both diluted in PBS containing 2% BSA, 0.3% Triton X-100 (antibody solution). After three PBS washes, cells were briefly re-blocked for 5 min and incubated for 90 min at RT with secondary antibodies: goat anti-mouse IgG Alexa Fluor 488 (Life Technologies, cat#A11029, 1:1000) and goat anti-rabbit IgG Alexa Fluor 546 (Life Technologies, cat#A11035, 1:1000), diluted in the same antibody solution. Following final PBS washes, coverslips were mounted using ProLong^™^ Glass Antifade Mountant with NucBlue^™^ (Invitrogen, cat#P36981). Images were acquired using a Zeiss Axio Imager Z1 microscope. For each condition, five random fields were acquired and analyzed using Fiji software (ImageJ).

### GFP-FLuc lentiviral vectors

Lentiviral vectors encoding GFP- firefly luciferase (GFP-Fluc) were produced in HEK293T cells as previously described [27] using the packaging plasmids psPAX2 (Addgene #12,260), pMD2G (Addgene, #12,259), and the transfer vector pHAGE PGK-GFP-IRES-LUC-W (Addgene #46,793) vectors. Human MBs were transduced at a multiplicity of infection (M.O.I) of 1 in the presence of polybrene (10 μg/ml) in GM. After overnight incubation, cells were washed and maintained in GM for an additional 3 days. GFP + myoblasts expressing the GFP-FLuc transgene were sorted by flow cytometry and expanded in GM until reaching 80% confluence. Cells were then switched to DM for 60 h. Following brief trypsinization and filtration through a 20 µm pre-separation filter, human MuRCs expressing the GFP-FLuc transgene (MuRC^GFP−FLuc^) were isolated.

### Mice and cell transplantation

Male immunodeficient NSG mice (NOD.Cg-Prkdcscid Il2rgtm1Wjl/SzJ, JAX:005557, The Jackson Laboratory), aged 10–12 weeks, were bred and housed at the University medical center animal facility, Switzerland. A total of 12 mice were anaesthetized with isoflurane (Abbott, Baar, Switzerland) and supplemented with oxygen via a semi-closed-circuit inhalation system. To induce muscle injury, both hind limbs were shaved and injected with 20 µl of cardiotoxin (CTX; 20 µM in 0.9% NaCl; Latoxan, France) into the gastrocnemius muscles one day prior to cell transplantation. Freshly isolated human MuRC-AF^High^ and MuRC-AF^Low^ cells (200,000 cells in 15 µl sterile PBS) were injected into the injured muscles using a 25 µl Hamilton syringe fitted with a 29G needle (Bonaduz, Switzerland). MuRC-AF^High^ cells were consistently injected into the left gastrocnemius, and MuRC-AF^Low^ cells into the right gastrocnemius of the same animal. Following transplantation, the skin was closed with single sutures using 5.0 resorbable thread. Postoperative analgesia was provided via subcutaneous injection of buprenorphine (Bupaq, 0.1 mg/kg in 0.9% NaCl) and continued in the drinking water (Bupaq, 0.01 mg/ml) for three days. For re-injury experiments (n = 6 mice), CTX was re-administered into the gastrocnemius muscle 28 days after the initial transplantation. Mice were euthanised by intraperitoneal injection of pentobarbital (150 mg/kg) either on day 28 (n = 6) or day 32 (n = 6) post transplantation.

### Bioluminescence imaging (BLI)

BLI was performed using an IVIS Spectrum system (PerkinElmer, Schwerzenbach, Switzerland) and data were analysed with Living Image® software (PerkinElmer). Mice received an intraperitoneal injection of luciferin (VivoGlow^™^ Luciferin, Promega, cat# P1041) at a dose of 100 mg/kg in PBS. Imaging was conducted over a 30-min period, consisting of ten consecutive 3-min acquisitions. For analysis, a constant region of interest (ROI) was applied across all samples and bioluminescence was quantified as total flux (photons/sec). Reference survival (set at 100%) was determined from the signal measured 3 h post-cell injection (day 0). BLI signals recorded on subsequent days were expressed as a percentage relative to the day 0 value.

### Immunocytochemistry

Harvested muscles were embedded in OCT compound (CellPath, Newtown, UK; cat#KMA-0100-00A) and snap-frozen in cold isopentane. Cryosections (10 μm thickness) were fixed 4% PFA in PBS (Sigma-Aldrich) for 15 min at RT, followed by PBS washes. Non-specific binding was blocked using PBS containing 5% goat serum (GS) and 0.3% Triton X-100 (Sigma-Aldrich) for 1 h at RT. Sections were incubated overnight at 4 °C with the following primary antibodies: mouse anti-human lamin A/C (human specific, 1:50, Invitrogen, cat# MA3-1000), mouse anti-Pax7 (1:20; DSHB, Iowa, USA) and rabbit anti-laminin (1:200, Abcam). After PBS washes, slides were incubated for 60 min at RT with Alexa Fluor-conjugated secondary antibodies (1:1000, Molecular Probes). Nuclei were counterstained and sections mounted using ProLongTM Glass Antifade Mountant with NucBlueTM (Invitrogen, cat#P36981). Images were acquired using an AxioScan Z1 microscope.

### Statistical analysis

All statistical analyses were performed using GraphPad Prism version 10.4 for MacOS. In vitro experiments included a minimum of four biological replicates and data are presented as mean ± SEM, with n indicating the number of independent experiments. Statistical comparisons were conducted using multiple unpaired Student’s t-tests, one-way ANOVA, or two-way ANOVA for comparisons involving more than two groups. When data did not meet normality assumptions (as assessed by normality tests), the non-parametric Wilcoxon–Mann–Whitney rank test was applied A p value < 0.05 was considered statistically significant (*p < 0.05, **p < 0.01, ***p < 0.001, ****p < 0.0001).

For the in vivo experiments, a total sample size of 12 mice per group was determined to be sufficient to detect a standardized effect size of 1.2 with 80% statistical power.

## Results

### Human MuRCs display higher levels of autofluorescence than human myoblasts

**Fig. 1:**
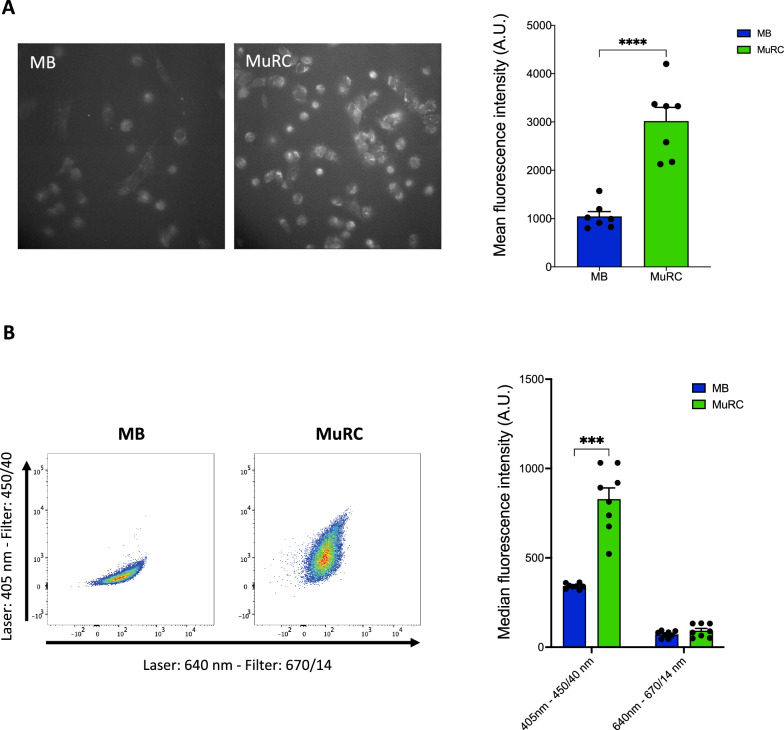
**Human MuRCs display higher autofluorescence than human MBs**. **A** Autofluorescence of freshly isolated human MuRCs and MBs was assessed by fluorescence microscopy using a 380 nm excitation and a 510/50 nm emission filter. The histogram presents the mean autofluorescence intensity (n = 7, mean ± SEM, multiple unpaired t-test; **** p < 0.0001). **B** Flow cytometry analysis of autofluorescence was performed on freshly isolated human MuRCs and MBs using various excitation/emission wavelengths. The histogram shows the median autofluorescence intensity (n = 8, mean ± SEM; multiple unpaired t-test; *** p < 0.001)

To compare autofluorescence (AF) levels between human myoblasts (MBs) and MuRCs, we first performed fluorescence microscopy using an excitation wavelength of 380 nm and an emission filter of 510/50 nm. MuRCs exhibited significantly higher AF intensity than MB, with a threefold increase in mean fluorescence intensity (Fig. 1A). Flow cytometry analysis further confirmed this difference: when excited at 405 nm and detected with a 450/40 bandpass filter (AF405), MuRCs showed a 2.4-fold increase in median AF intensity compared to MBs (Fig. 1B). Notably, MuRCs also displayed greater heterogeneity in AF signal distribution under these conditions (Fig. 1B).

### Autofluorescence correlates with increased lipid content in human MuRCs

**Fig. 2:**
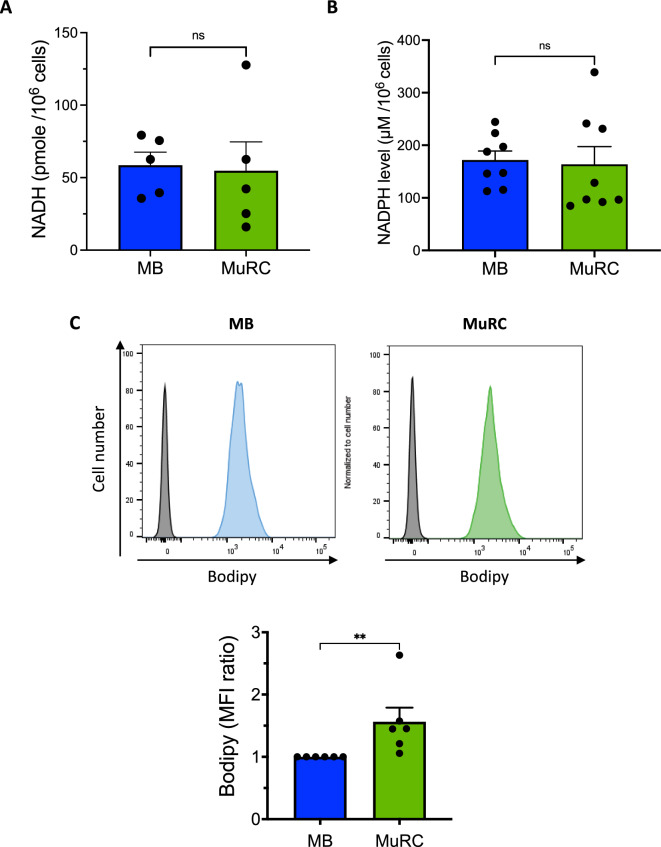
**Autofluorescence correlates with increase lipid content in human MuRCs**. Freshly isolated human MuRCs and MBs were lysed to measure intracellular levels of NADH (**A**) and NADPH (**B**) using specific enzymatic assays. Data are shown as mean ± SEM, with statistical analysis performed using the Mann–Whitney test (n = 5 for A) and unpaired t-test (n = 9 for B). For lipid droplet quantification (C), cells were labelled with BODIPY for 15 min, washed and analysed by flow cytometry. The histogram displays the mean fluorescence intensity (MFI) ratio of BODIPY staining relative to MBs (n = 6, mean ± SEM; Mann–Whitney test; ** p < 0.001)

Cellular AF covers a broad spectral range due to the presence of multiple endogenous fluorophores, each emitting at distinct wavelengths. For instance, when excited at appropriate wavelengths, flavins, NADPH and lipofuscin emit green, blue and orange fluorescence respectively [21]. To identify potential contributors to the AF signal observed upon 450 nm excitation, we examined several candidate molecules including NADH, NADPH and fatty acids [25]. Colorimetric quantification revealed no significant differences in NADH and NADPH levels between human MBs and MuRCs (Fig. 2A and B). To assess lipid accumulation, we stained cells with the BODIPY fluorophore and performed flow cytometry. This analysis showed a 1.6-fold increase in lipid content in human MuRC compared to MBs (Fig. 2C), suggesting that intracellular lipids may contribute to the elevated AF signal observed in MuRC.

### High autofluorescent human MuRC are enriched in Pax7^High^ cells.

**Fig. 3:**
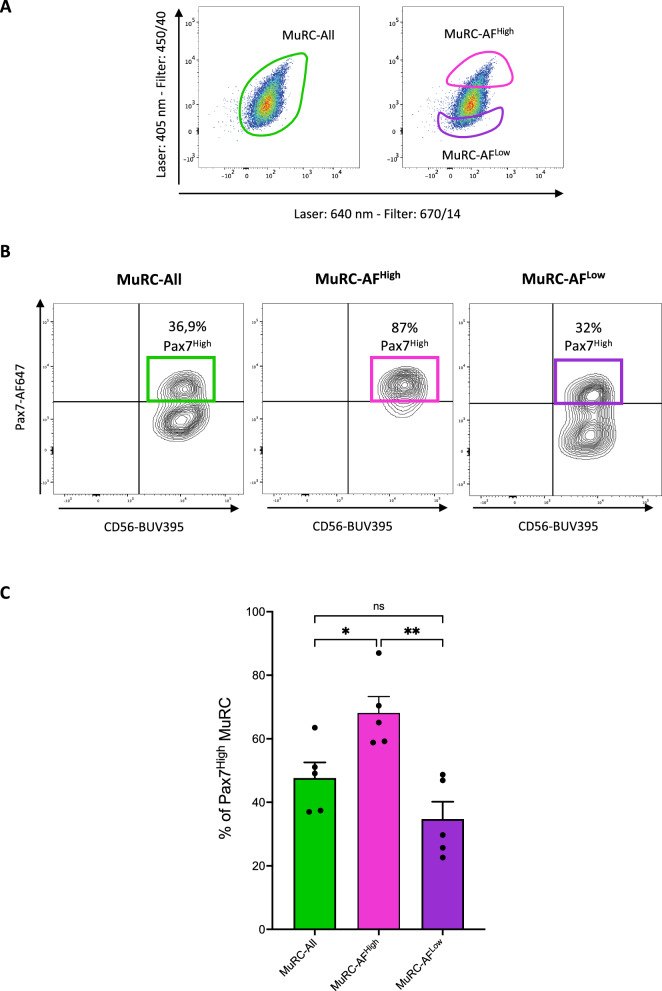
**Human MuRC with high autofluorescence (MuRC-AF**^**High**^**) represent a Pax7**^**High**^**-enriched subpopulation**. **A** Human MuRCs were sorted by flow cytometry based on their autofluorescence signal: the top 10% with the highest autofluorescence (MuRC-AF^High^), the bottom 10% with the lowest autofluorescence (MuRC-AF^Low^) and the total MuRC population (MuRC-All). **B** Sorted cells were fixed, permeabilized, and co-stained for Pax7 and CD56, followed by flow cytometry analysis. **C** The percentage of Pax7^High^ cells within each MuRC subpopulations is shown. Data are presented as mean ± SEM (n = 5); statistical analysis was performed using one-way ANOVA (*p < 0.05; **p < 0.01

When excited at 405 nm and detected using a 450/40 bandpass filter, human MuRCs exhibited significantly higher AF compared to human MBs. To investigate the relationship between AF levels and Pax7 expression, we used flow cytometry to sort human MuRC into three groups based on AF levels: MuRC-All (entire population), MuRC-AF^High^ (top 10% highest AF signal) and MuRC-AF^Low^ (bottom 10% lowest AF signal) (Fig. 3A). Freshly isolated human MuRC were immediately stained for Pax7 and CD56 and analysed by flow cytometry. The MuRC-AF^High^ population contained a significantly higher proportion of Pax7^High^ cells (68 ± 5.1%) compared to MuRC-All (47 ± 4.9%) and MuRC-AF^Low^ (34.7 ± 5.4%) (Fig. 3B and C). Despite this enrichment, we observed notable biological variability in the proportion of Pax7^High^ cells within the MuRC-AF^High^ population, ranging from 58 to 87% across biological replicates.

### Human MuRC-AF^High^ require more time to reactivate and form smaller colonies

**Fig. 4:**
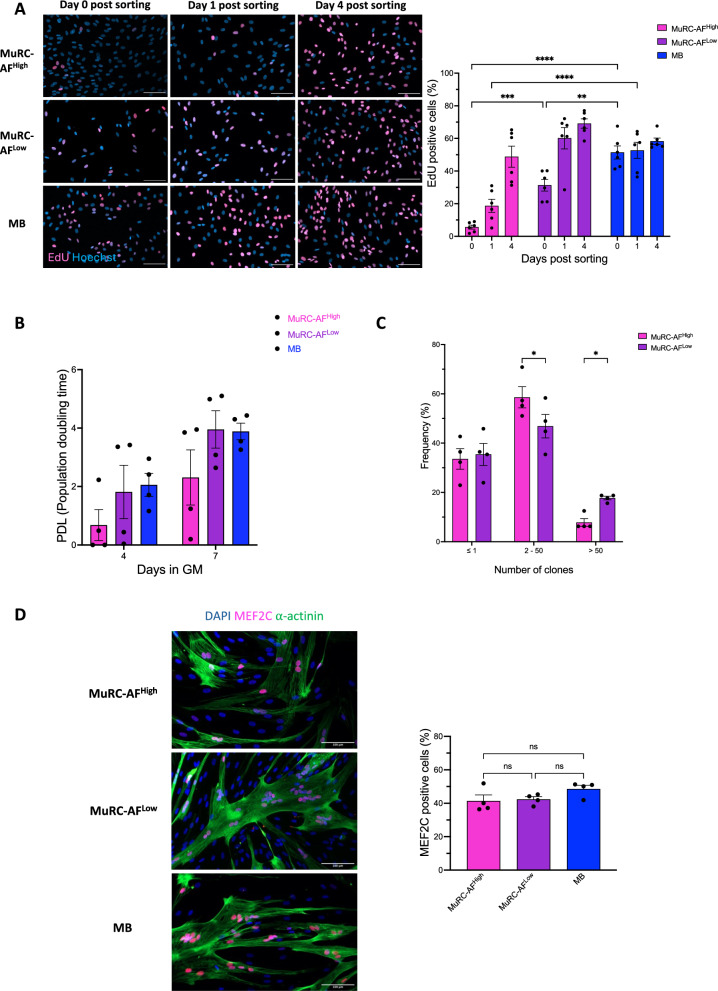
**Human MuRC-AF**^**High**^** require more time to reactivate, form smaller colonies, and differentiate efficiently into myotubes**. Freshly isolated human MB, MuRC-AF^High^ and MuRC-AF^Low^ cells were sorted by flow cytometry. **A** Cells were cultured in GM containing EdU for 24 h, starting either on the day of sorting (D0), one day after sorting (D1) or four days after sorting (D4). Representative images show EdU incorporation (magenta) and Hoechst nuclear staining (blue). The histogram displays the percentage of EdU-positive cells (n = 6, mean ± SEM; two-way ANOVA; ** p < 0.01; *** p < 0.001; ****p < 0.0001). **B** A total of 30,000 freshly isolated cells were seeded and cultured in GM. Cell counts were performed on days 4 and 7 post sorting. Histograms shoe population doubling level (PDL) from four independent experiments (n = 4, one-way ANOVA; ns > 0.05). **C** Clone size distribution was quantified from four independent experiments, each analyzing 96 single cells. Data are presented as mean ± SEM (n = 4, two-way ANOVA; * p < 0.05. **D** Cells were cultured in DM for 48 h and immunostaied for α-actinin (green), MEF2C (magenta) and DAPI (blue). The percentage of MEF2C-positive nuclei was quantified (n = 4, mean ± SEM; Kruskal–Wallis test)

Freshly isolated human MB, MuRC-AF^High^, and MuRC-AF^Low^ cells were sorted by flow cytometry to assess their cell cycle re-entry kinetics. Cells were cultured in GM with EdU for 24 h, starting at day 0 (D0), day 1 (D1) or day 4 (D4) post sorting. At D0, MuRC-AF^High^ showed minimal EdU incorporation (5.6 ± 1.2%), significantly lower than MuRC-AF^Low^ (31.3 ± 3.7%) and MBs (51.4 ± 4%) (Fig. 4A). This delayed activation persisted at D1 with only 18.7 ± 4% EdU-positive MuRC-AF^High^ (Fig. 4A). By D4, EdU incorporation was comparable across all groups: 48.7 ± 6% (MuRC-AF^High^), 69.1 ± 3% (MuRC-AF^Low^) and 58.2 ± 2% (MBs) (Fig. 4A). While population doubling level (PDL) did not differ significantly, MuRC-AF^High^ showed a trend towards reduced proliferation (Fig. 4B). Clonal efficiency was also assessed by seeding single MuRC-AF^High^ and MuRC-AF^Low^ cells in 96-well plates and culturing them in GM for eight days. Both populations exhibited similar clonal efficiency, with around 65% of wells containing more than one nucleus (Fig. 4C). However, MuRC-AF^Low^ colonies were larger with 17.7% containing more than 50 nuclei, compared to only 7.8% in MuRC-AF^High^ (Fig. 4C). Finally, we evaluated the myogenic differentiation potential of each population. Freshly isolated MBs, MuRC-AF^High^ and MuRC-AF^Low^ cells were seeded at high density in GM for 24 h, then switched to DM for 48 h. All groups efficiently formed multinucleated myotubes, with comparable percentage of MEF2C-positive nuclei: 48.6% (MBs), 41.4% (MuRC-AF^High^) and 42.3% (MuRC-AF^Low^) (Fig. 4D).

### GFP-FLuc transduction preserves autofluorescence in human MuRCs.

**Fig. 5:**
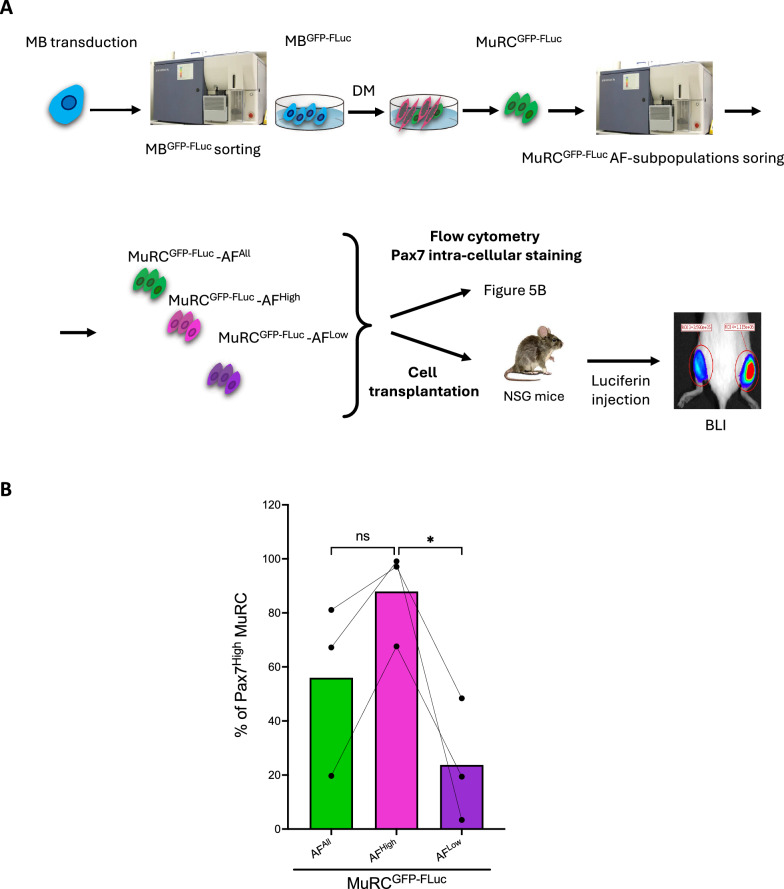
**Generation of human MuRCs expressing the GFP-FLuc transgene**. **A** Schematic overview of the experimental strategy used to generate human MuRCs expressing the GFP-firefly luciferase (FLuc) transgene. Human MuRC^GFP−FLuc^ were sorted by flow cytometry based on their autofluorescence signal prior to either transplantation into immunodeficient mice or intracellular Pax7 staining. **B** Freshly isolated human MuRC^GFP−FLuc^ subpopulations (AF^All^, AF^High^ and AF^Low^) were immediately stained for Pax7 and analysed by flow cytometry. The percentage of Pax7^High^ cells within each MuRC subpopulations is shown. Data are presented as mean ± SEM (n = 3, Kruskal–Wallis; * p < 0.05)

Bioluminescence imaging (BLI) provides a non-invasive and quantitative method to monitor the survival of human MuRCs following transplantation. To enable BLI, human MBs were transduced with a lentiviral vector encoding GFP and firefly luciferase (Fluc). After three days in GM, CD56 + /GFP-FLuc + MBs were isolated by flow cytometry, expanded to 80% confluence, and then differentiated in vitro for 60 h. Human MuRCs expressing the GFP-FLuc transgene (MuRC^GFP−FLuc^) were subsequently isolated using AF405/AF647 gating to define AF^All^, AF^High^ and AF^Low^ subpopulations. These cells were stained for Pax7 and analysed by flow cytometry (Fig. 5A). The MuRC^GFP−FLuc^-AF^All^ population displayed a similar proportion of Pax7^High^ cells compared to untransduced MuRC-AF^All^ (Fig. 5B and 3C). Furthermore, the MuRC^GFP−FLuc^-AF^High^ subset showed a higher percentage of Pax7^High^ cells than the MuRC^GFP−FLuc^-AF^Low^ counterpart (Fig. 5B). These findings indicate that GFP-FLuc transduction does not alter the AF properties or Pax7 expression profile of human MuRCs.

### Regenerative capacity of human MuRC-AF^High^ and MuRC-AF^Low^ following transplantation into immunodeficient mice

**Fig. 6:**
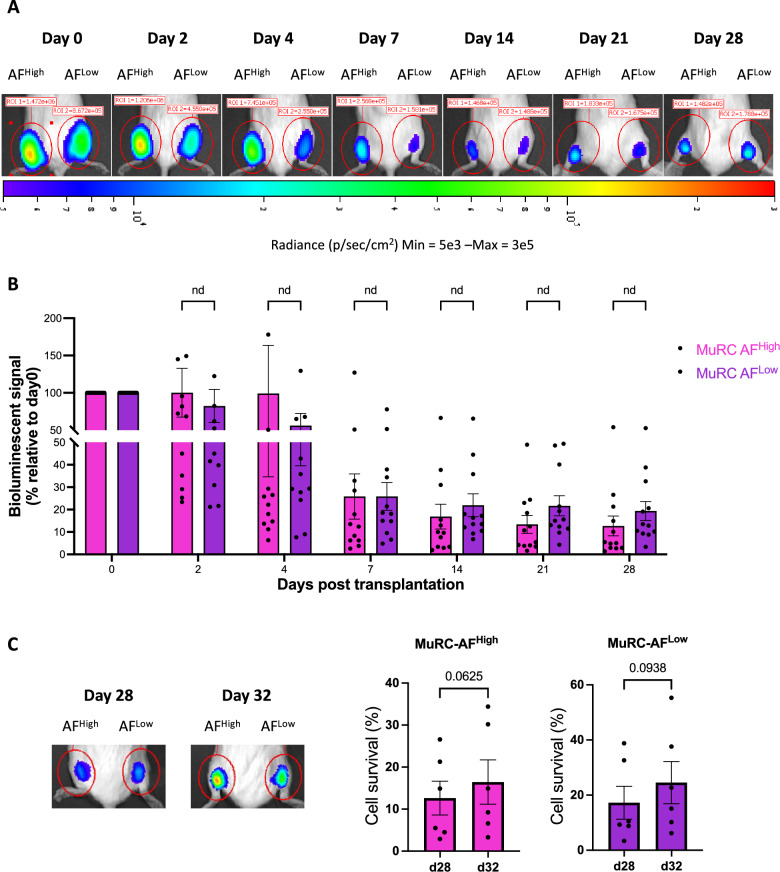
**Cell survival following transplantation of AF**^**High**^** or AF**^**Low**^** human MuRCs into injured muscles of immunodeficient mice**. **A** Representative bioluminescence images showing the time course of cell survival after transplantation of 200,000 MuRC-AF^High^ (left Gastrocnemius) or MuRC-AF^Low^ (right Gastrocnemius) into injured muscles. **B** Quantification of cell survival over time. Survival was normalized to the BLI signal measured 3 h post-injection (day 0), defined as 100% survival for each injected muscle. Data are presented as mean ± SEM (n = 12, multiple unpaired t-test, ns > 0.05). **C** Human MuRCs retain functionality as muscle stem cells in vivo. Gastrocnemius muscles of six mice were re-injured with cardiotoxin 28 days after initial transplantation. The histogram shows the percentage of surviving cells 4 days after re-injury (day 32). Data are presented as mean ± SEM (n = 6, Wilcoxon test; * p < 0.05)

To evaluate the in vivo regenerative potential of human MuRC-AF^High^ and MuRC-AF^Low^ subpopulations, freshly isolated MuRC^GFP−FLuc^ (AF^High^ and AF^Low^) were transplanted into cardiotoxin-injured gastrocnemius muscles of immunodeficient mice. Cell survival was monitored non-invasively using BLI over a 28-day period (Fig. 6A and B). There was no difference in the percentage of surviving human MuRCs between the AF^High^ and AF^Low^ subpopulations at any of the analysed time point (Fig. 6B). Both populations exhibited substantial cell loss by day 7, with survival rates dropping to approximately 25% in each group (Fig. 6B). By day 28, survival further declined to 12.7% for MuRC-AF^High^ and 19.4% for MuRC-AF^Low^, with no statistically significant difference between them (Fig. 6B).

**Fig. 7:**
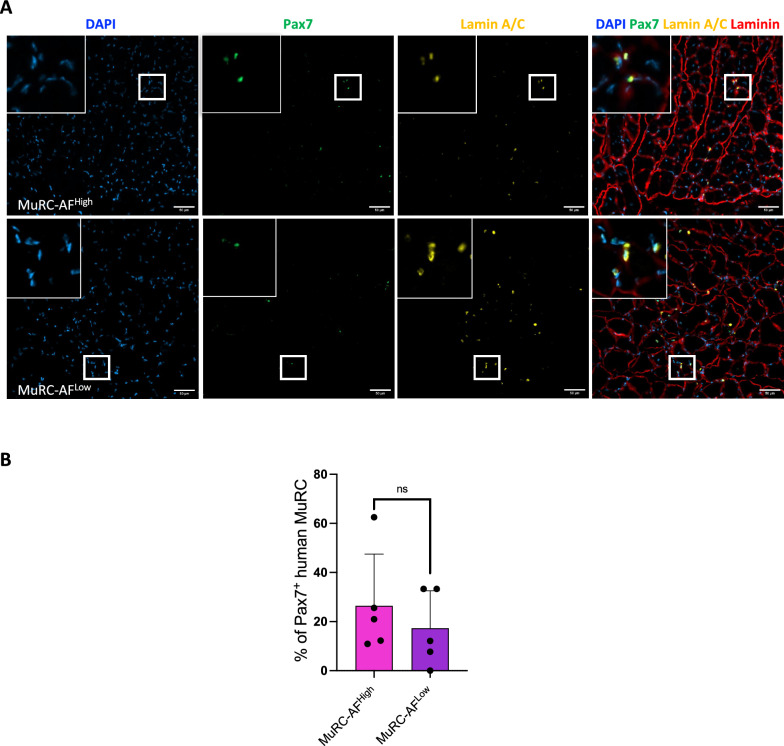
**Histological analysis following transplantation of human MuRC-AF**^**High**^** and AF**^**Low**^** into injured muscles**. **A** Twenty-eight days post-transplantation, muscle sections were triple stained with antibodies against Pax7 (green), human lamin A/C (orange) and laminin (red). Nuclei were counterstained with DAPI (blue). Cells positive for Pax7 and human lamin A/C were detected in all muscles injected with either MuRC-AF^High^ or MuRC-AF^Low^. **B** Quantification of Pax7 + cells among human MuRCs (expressed as the percentage of Pax7 + cells per total number of lamin A/C + cells) in representative sections. Data are presented as mean ± SEM (n = 5, unpaired Student's t-test, p > 0.05)

To assess the capacity of transplanted cells to respond to a secondary injury, muscles were re-injured with cardiotoxin on day 28, and BLI was performed four days later (Fig. 6C). A modest increase in bioluminescence signal was observed in both groups, suggesting reactivation of surviving cells: from 12.6% to 16.4% in MuRC-AF^High^ and from 17.2% to 24.5% in MuRC-AF^Low^ (Fig. 6C). Although not statistically significant, these results indicate that both subpopulations retain the ability to respond to a regenerative cue. Immunohistological analysis further confirmed the contribution of transplanted MuRCs to muscle regeneration. Co-staining for human lamin A/C, Pax7, and laminin revealed that both MuRC-AF^High^ and MuRC-AF^Low^ contributed to the formation of human-derived MuSCs. Specifically, 26.4 ± 9.4% of human lamin A/C⁺ cells in the MuRC-AF^High^ population were Pax7 + compared to 18.2 ± 6.7% in the MuRC-AF^Low^ group (Fig. 7).

## Discussion

MuSCs hold therapeutic promise for treating muscle diseases, however their clinical potential is limited by rapid activation and subsequent loss of regenerative capacity under standard culture conditions [9, 10]. We previously identified human MuRCs as a population of quiescent Pax7 + MuSCs with enhanced therapeutic potential, particularly a Pax7^High^ subpopulation in a deeper quiescent state [16]. Despite their promise, current methods for distinguishing human Pax7^High^ and Pax7^Low^ subpopulations rely on intracellular flow cytometry staining, which compromises cell viability and precludes downstream functional analyses both in vitro and in vivo.

This study demonstrates that human MuRCs exhibit significantly higher levels of intrinsic AF compared to human MBs, indicating fundamental differences in their biochemical composition. Fluorescence microscopy and flow cytometry revealed a threefold increase in mean fluorescence intensity in MuRCs, with the most pronounced AF observed under 405 nm excitation and 450/40 nm emission detection. The absence of significant signal at 640 nm suggests that the contributing endogenous fluorophores primarily emit within the lower wavelength range [21]. Given that AF is commonly attributed to cellular components such as flavins, NADPH and lipofuscin, we investigated the biochemical basis of this signal. Quantitative assays showed no significant differences in NADH and NADPH levels between MBs and MuRCs, indicating these cofactors are unlikely to account for the elevated AF. In contrast, flow cytometric analysis using BODIPY staining revealed a 1.6-fold increase in lipid content in MuRCs suggesting that lipid accumulation may contribute to their heightened AF. This is consistent with previous reports showing that intracellular lipids, particularly in the form of lipid droplets, possess fluorescent properties when excited at specific wavelength [28, 29]. Moreover, our prior transcriptomic analysis supports this observation [16], revealing that human MuRCs are enriched in genes associated with fatty acid oxidation (FAO) and display reduced expression of glycolytic genes. This metabolic profile aligns with the well-established shift from glycolysis to FAO observed in quiescent MuSCs [30].

Further characterisation of MuRCs based on their AF intensity reveals that the MuRC-AF^High^ subset constitutes a distinct subpopulation enriched in Pax7^High^ cells, a well-established marker of muscle stemness. The observed heterogeneity in AF signals within the human MuRC population suggests that AF may serve as a valuable biomarker for identifying functionally distinct muscle stem/progenitor cells subsets [31]. Notably, the MuRC-AF^High^ group exhibited greater variability in Pax7 expression across biological replicates, indicating potential influences from donor-specific factors or intrinsic cellular heterogeneity within the MuRC population.

Functional assays revealed that MuRC-AF^High^ cells require more time to exit quiescence and re-enter the cell cycle compared to MuRC-AF^Low^ and MB populations. This delayed activation is consistent with previous findings showing that quiescent MuSCs exhibit reduced metabolic activity and prolonged cell cycle re-entry [17, 32, 33]. Although population doubling levels were comparable across groups, MuRC-AFHigh cells displayed a trend toward lower proliferative capacity. Clonal expansion assays further supported this observation: while both MuRC subpopulations showed similar cloning efficiency, MuRC-AF^High^ colonies contained fewer nuclei, indicating a slower proliferation rate. These findings suggest that MuRC-AF^Low^ cells, characterized by lower Pax7 expression, may possess higher proliferative and engraftment potential following intramuscular transplantation, paralleling observations in murine MuSCs, where lower Pax7 expression correlates with enhanced regenerative performance in vitro [34]. Despite differences in proliferation dynamics, all three populations (MB, MuRC-AF^High^ and MuRC-AF^Low^) retained robust myogenic differentiation capacity, forming multinucleated myotubes with similar efficiency. Importantly, we also demonstrated that MuRCs transduced with GFP-FLuc maintain their AF properties and preserve the distribution of Pax7^High^ cells within both AF^High^ and AF^Low^ subsets. This confirms that transgene expression does not interfere with key human MuRC characteristics and supports the use of bioluminescence imaging (BLI) as a reliable, non-invasive tool to track MuRC behavior in vivo following transplantation. We also observed significant cell loss by day seven post injection, indicating a low survival rate of human MuRCs in vivo. This may be partially attributed to the use of flow cytometry for isolating AF^High^ and AF^Low^ MuRCs, a process known to induce cellular stress and potentially compromise viability and function [35, 36]. Interestingly, both subpopulations showed increased proliferation following re-injury, with no significant differences between the groups. Consistent with findings in other myogenic progenitor cell studies [7, 17, 37, 38], our results provide the first evidence that both human MuRC-AF^High^ and -AF^Low^ subpopulations function as bona fide MuSC in vivo, retaining the capacity to reactivate in response to injury. Immunohistological analysis confirmed their contribution to the MuSC pool, with MuRC-AF^High^ cells showing a slightly higher proportion of Pax7 + cells populations within the human lamin A/C⁺ population compared to MuRC-AFLow cells. These findings suggest that, despite their delayed activation and reduced proliferative capacity, MuRC-AF^High^ cells maintain regenerative potential and can contribute to muscle repair upon secondary injury.

Importantly, the use of AF as a biomarker provides a powerful and non-invasive strategy for isolating functionally distinct subpopulations of human MuRCs without compromising cell viability or cell function. Unlike conventional intracellular staining methods, which require cell fixation and permeabilization, AF-based sorting enables the isolation of human MuRC subpopulations based on their intrinsic fluorescence properties. This preserves their regenerative potential for use in subsequent applications. This method supports the development of clinically compatible protocols for the isolation of human muscle stem cells, enabling real-time cell selection and transplantation without the need for genetic modification or antibody labeling. Therefore, AF-based identification is a valuable tool for advancing the therapeutic use of muscle stem cells in regenerative medicine.

## Conclusion

Our study demonstrates that human MuRCs exhibit significantly higher levels of AF compared to human myoblasts (MBs), with AF correlating with increased intracellular lipid content. Within the MuRC populations, AF^High^ cells are enriched in Pax7^High^ cells and display delayed cell cycle re-entry and slower proliferation. Despite these differences, MuRC-AF^High^ cells retain robust myogenic differentiation and regenerative capacity. These findings position AF as a promising label-free biomarker for identifying functionally distinct subsets of human muscle progenitor cells and underscore its potential utility in muscle regeneration research. Future studies should investigate the metabolic implications of lipid accumulation in human MuRCs and its impact on muscle stem cell function, particularly in the contexts of aging and muscle disease.

## Data Availability

The datasets used and/or analyzed during the current study are available from the corresponding author on reasonable request.

## Abbreviations

AF: Autofluorescence
BLI: Bioluminescence imaging
CTX: Cardiotoxin
DM: Differentiation medium
FAO: Fatty acid oxidation
GM: Growth medium
GS: Goat serum
MB: Myoblast
MOI: Multiplicity of injection
MuSC: Muscle stem cell
MuRC: Muscle reserve cell
RT: Room temperature
